# Synthesis and selected immunological properties of 10-substituted 1,8-diazaphenothiazines

**DOI:** 10.1007/s00044-014-1220-9

**Published:** 2014-08-19

**Authors:** Beata Morak-Młodawska, Krystian Pluta, Michał Zimecki, Małgorzata Jeleń, Jolanta Artym, Maja Kocięba

**Affiliations:** 1 Department of Organic Chemistry, The Medical University of Silesia, Jagiellońska 4, 41-200 Sosnowiec, Poland; 2 Department of Experimental Therapy, Institute of Immunology and Experimental Therapy, Polish Academy of Sciences, R. Weigla 12, 53-114 Wrocław, Poland

**Keywords:** Phenothiazines, Diazaphenothiazines, Antiproliferative activity, Anticancer activity, Thiazine ring formation

## Abstract

A new type of tricyclic azaphenothiazines—1,8-diazaphenothiazines—was obtained in the reaction of 2,3- and 3,4-disubstituted pyridines. The reaction ran as the Smiles rearrangement. The 1,8-diazaphenothiazine system was determined using NOE experiment and 2D NMR spectra (COSY, HSQC, HMBC). 10*H*-1,8-diazaphenothiazine was transformed into 10-derivatives with alkyl, aminoalkyl, amidoalkyl, sulfonamidoalkyl, and nitrogen half-mustard groups. The compounds were tested for their effects on phytohemagglutinin A-induced proliferative response of human peripheral blood mononuclear cells (PBMC) and lipopolysaccharide-induced tumor necrosis factor alpha production by human whole blood cultures. The compounds exhibited differential, dose-dependent inhibitory activities in these tests. All the compounds were low toxic against PBMC. The compounds showing the highest antiproliferative activity strongly inhibited the growth of leukemia L-1210 and colon cancer SW-948 cell lines, similarly as cisplatin, a reference drug.

## Introduction

Tricyclic phenothiazines attract considerable attention because of their significant biological activities and interesting chemical features. Classical phenothiazines with aminoalkyl substituents at the nitrogen atom are the source of valuable drugs exhibiting neuroleptic, antihistaminic, antitussive, and antiemetic activities (Gupta and Kumar, 1988). The structure modifications of these compounds were carried out by introduction of new substituents, mainly at the thiazine nitrogen atom, and substitution of one or two benzene rings with homoaromatic and heteroaromatic rings. The modifications with azine rings lead to formation of azaphenothiazines. New phenothiazines can contain not only the tricyclic ring system but also tetra and pentacyclic ones with up to four additional nitrogen atoms in the aromatic rings (Silberg *et al.,* 2006; Pluta *et al.,* 2009, 2011). Such modifications can change potency and type of activities of the basic structures. Recent reports describe very promising anticancer, antibacterial, and anti-inflammatory activities, reversal of multidrug resistance and a potential benefit in treatment of Alzheimer’s, Creutzfeldt-Jakob’s and AIDS-associated diseases for the modified phenothiazines (Motohashi *et al.,* 2000, 2006; Dasgupta *et al.,* 2008; Sadandam *et al.,* 2009; Aaron *et al.,* 2009; Tandon *et al.,* 2009; Pluta *et al.,* 2011).

Our strategy for modification of the phenothiazine structure is based on the introduction of two pyridine rings instead of the benzene ones to form dipyrido[1,4]thiazines. Among ten theoretically possible dipyridothiazines types only four have been known before introduction of our research strategy, i.e., 1,6- (Maki, 1957; Takahashi and Maki, 1958a, b; Rodig *et al.,* 1966), 1,9- (Rath, 1957), 2,7- (Kopp *et al.,* 1963; Kopp and Strell, 1962), and 3,6-diazaphenothiazines (Okafor, 1967). Three nomenclature systems of phenothiazines with different atom numbering, valid in the sixties and seventies, were confusing. 2,7-Diazaphenothiazines described by Kopp and co-workers were in fact 3,7-diazaphenothiazines (Pluta *et al.,* 2009). Correct 2,7-diazaphenothiazines were obtained by us and their ring system was confirmed by X-ray analysis (Morak *et al.,* 2002; Morak and Pluta, 2007). The parent compound, 10*H*-2,7-diazaphenothiazine, was found to be a universal, low-toxic immunosuppressant, inhibiting both humoral and cellular immune responses, and antioxidant property (Zimecki *et al.,* 2009; Morak-Młodawska *et al.,* 2010; Pluta *et al.,* 2010).

In continuation of our studies, we have worked out an efficient synthesis of a new type of dipyridothiazines, 10*H*-1,8-diazaphenothiazine and its 10-substituted derivatives, possessing alkyl, arylalkyl, aryl, heteroaryl and aminoalkyl, amidoalkyl, sulfonamidoalkyl, and nitrogen half-mustard type substituents. In this work, we discuss their synthesis and structures and test their activities in selected biological assays.

## Results and discussion

### Chemistry

It is well known that the synthesis of phenothiazines and azaphenothiazines may proceed via cyclization of diphenyl sulfides, phenyl azinyl sulfides, or diazinyl sulfides directly as the Ullmann cyclization or with the Smiles rearrangement of the S ***→*** N type depending on the reaction conditions. In the last case, the phenyl or azinyl part migrates from the sulfur atom to the nitrogen atom forming amine and subsequently phenothiazine or azaphenothiazine. The rearrangement proceeds most often under basic but also under acidic and neutral conditions. Sometimes it is impossible to state if a reaction runs with or without the rearrangement because the Ullmann and Smiles products are the same or very similar (Pluta *et al.,* 2009).

**Scheme 1:**
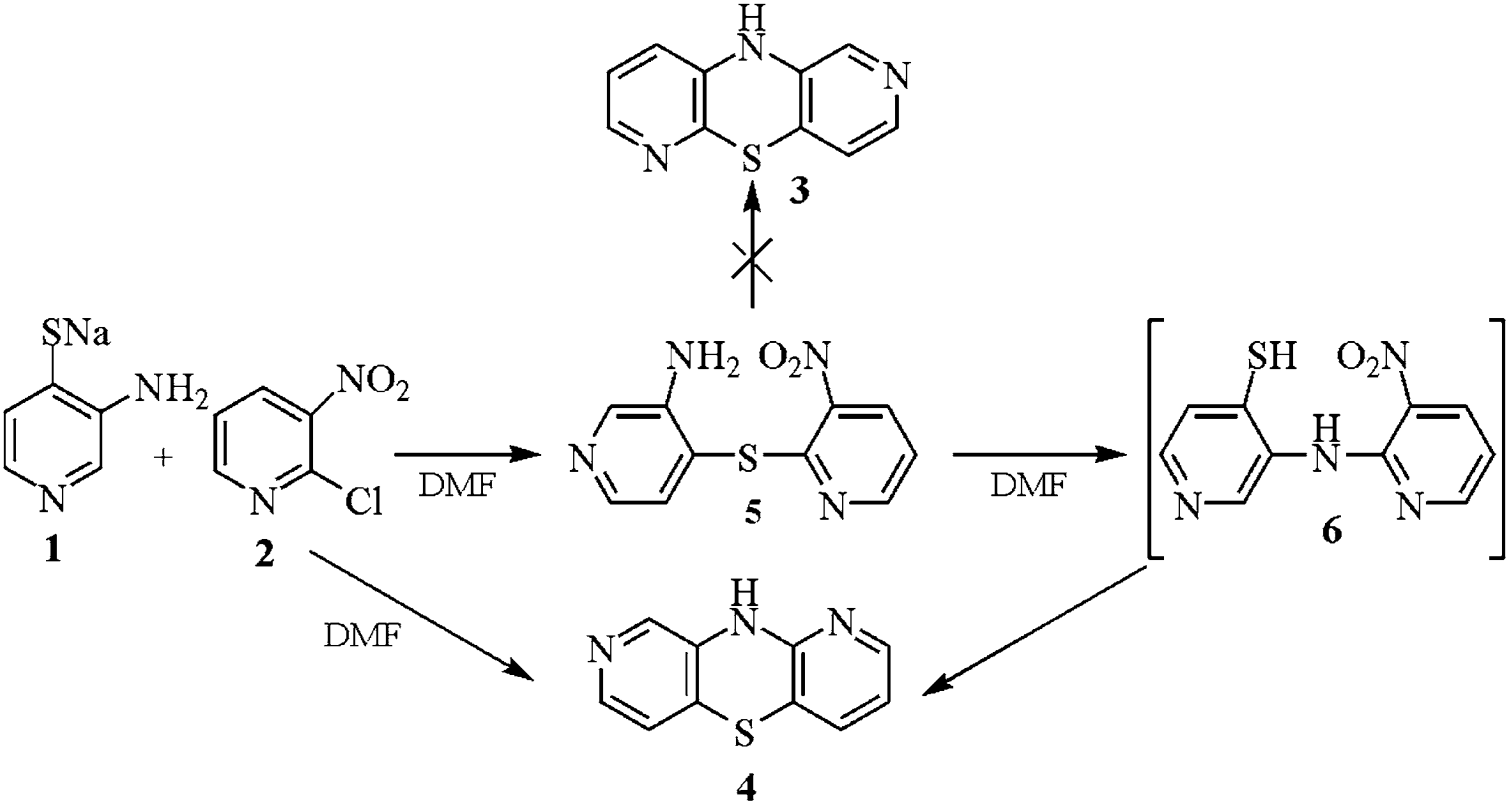
Synthesis if 10*H*-diazaphenothiazine **3** from disubstituted pyridines **2** and **3** and dipyridyl sulfide **5**

**Scheme 2:**
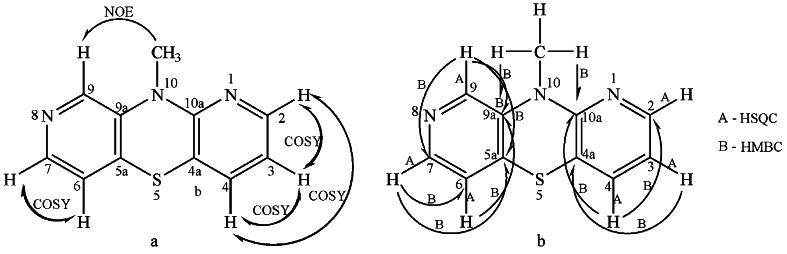
The NMR experiments for compound **7**: *a* NOE and COSY, *b* HSQC and HMBC

**Scheme 3:**
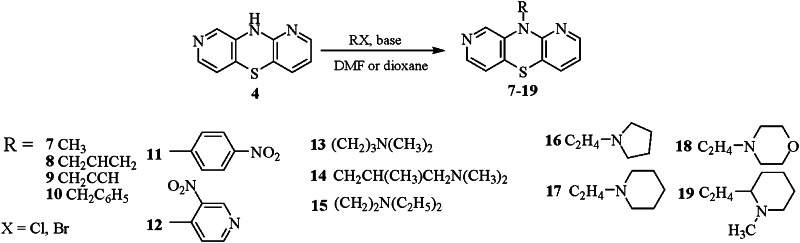
Synthesis of 10-dialkylaminoalkyl-1,8-diazaphenothiazines **7**–**19**

We started the synthesis with a reaction of sodium 3-aminopyridinothiolate (**1**) with 2-chloro-3-nitropyridine (**2**) in refluxing DMF. After isolation and purification of the products we found dipyridothiazine (2,6-diazaphenothiazine **3** or 1,8-diazaphenothiazine **4**) as the major product in 88 % yield and 3′-amino-3-nitro-2,4′-dipyridyl sulfide (**5**) in 9 % yield as the minor product (Scheme 1). The mass spectrum confirmed the diazaphenothiazine structure (*M* = 201) but the ^1^H NMR spectrum does not point at the structure **3** or **4** as both compounds are built of the 2,3- and 3,4-pyridinediyl units giving a singlet (7.90 ppm), two doublets (7.18, 8.07 ppm), and three doublets of doublet (6.90, 7.26, 8.09 ppm) of the proton signals. To unquestionably determine the diazaphenothiazine structure, we transformed the product into the N-methyl derivative (vide infra). The differentiation between 1,8- and 2,6-diazaphenothiazine system was based on the NOE experiment of this derivative. Irradiation of the methyl protons at 3.44 ppm (Scheme 2) gave an enhancement only of one proton, the singlet signal at 7.90 ppm by 7.06 % what pointed at the 1,8-diazaphenothiazine system and the derivative **7** (Scheme 3).

The full ^1^H NMR assignment of the proton signals came from the homonuclear ^1^H–^1^H correlation (COSY). Three most deshielded proton signals at 7.90, 8.07, and 8.09 ppm were considered as the α-pyridinyl proton signals. The doublet of doublet signal at 6.90 ppm, considered as the β-pyridinyl proton, was intercorrelated (*ortho*-coupling) with the signals at 8.09 ppm and at 7.26 ppm (γ-pyridinyl proton) with the coupling constants of 4.9 and 7.2 Hz, respectively. The signal at 7.26 ppm was weak intercorrelated (*para*-coupling) with the signal at 8.09 ppm with the coupling constant of 1.8 Hz. The protons were assigned as H_3_, H_4_, and H_2_, respectively. The α-pyridinyl proton signal at 8.07 ppm was correlated with the signal at 7.18 ppm (β-pyridinyl proton) with the coupling constant of 5.4 Hz. These protons were assigned as H_7_ and H_6_. The proton signal assignment was presented in Scheme 2.

The new diazaphenothiazine system was also determined by the ^13^C NMR spectrum. The spectrum revealed eleven carbon signals: one primary, six tertiary, and four quaternary. The methyl group was observed at 32.8 ppm. The full assignment of carbon signals came from 2D NMR: HSQC (the tertiary carbon atoms connected with the hydrogen atoms) and HMBC (the tertiary and quaternary carbon atoms correlated with the hydrogen atoms via two and mainly three bonds). The proton-carbon correlation was presented in Scheme 2.

The product structure as 10*H*-1,8-diazaphenothiazine **4** is the evidence for the Smiles rearrangement of the S–N type of resulted dipyridinyl sulfide **5**. Heating sulfide **5** in refluxing DMF gave 10*H*-1,8-diazaphenothiazine (**4**) in 88 % yield. The reaction run through the formation of dipyridinyl amine **6** which (not isolated) very easily cyclized to diazaphenothiazine **4** (Scheme 1). The 1,8-diazaphenothiazine ring system was confirmed by X-ray analysis of the nitropyridyl derivative **12** (obtained by independent way from appropriate sulfide containing three nitropyridyl moieties via the double Smiles rearrangement), published separately (Morak-Młodawska *et al.,* 2012).

The parent 10*H*-1,8-diazaphenothiazine **4** was transformed into 10-derivative in one or three steps. The alkylation with alkyl (methyl, allyl, propargyl, benzyl), aryl (p-nitrophenyl) and heteroaryl (3-nitro-4-pyridinyl) halides and aminoalkyl (3-dimethylaminopropyl, 3-dimethylamino-2-methylpropyl, 2-diethylaminoethyl, 1-pyrrolidinoethyl, 1-piperidinoethyl, 1-methyl-2-piperidinoethyl, 1-morpholinoethyl) in DMF in the presence of sodium hydride or potassium *tert*-butoxide and in dioxane in the presence of sodium hydroxide gave derivatives **7**–**19** in good yields Scheme 3).

**Scheme 4:**
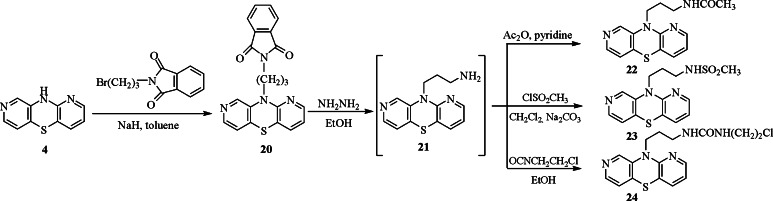
Synthesis of 10-phthalimidopropyl-1,8-diazaphenothiazine **20** and transformations to the acetamidopropyl, methanesulfonamidopropyl, and chloroethylureidopropyl derivatives **22**–**24**

The substrate **4** was also transformed into compounds possessing aminopropyl derivative substituents. Reaction of compound **4** with the phthalimidopropyl bromide in toluene in the presence of sodium hydride gave the phthalimidopropyl derivative **20**. The hydrolysis of this compound with hydrazine in ethanol led to aminopropyl derivative **21** which quickly (because of their instability) underwent reactions with acetic anhydride, methanesulfonyl chloride, and 2-chloroethyl isocyanate to give acetamidopropyl, methanesulfonamidopropyl, and chloroethylureidopropyl derivatives **22**–**24** in 63–80 % yield (Scheme 4).

### Biological activities

10-substituted 1,8-diazaphenothiazines **4**, **7**–**10**, **12**–**20,** and **22**–**24**, possessing various substituents (hydrogen atom, alkyl groups with single, double, and triple bonds, arylalkyl, heteroaryl, alkylaminoalkyl, amidoalkyl, sulfonamidoalkyl and alkyl with a half-mustard-type group) were tested for their biological activities. The tests included the proliferative response of human peripheral blood mononuclear cells (PBMC) induced by phytohemagglutinin A (PHA), the cytotoxic effect on human PBMC and lipopolysaccharide (LPS)-induced production of tumor necrosis factor alpha (TNF-α). The combined results of the tests are presented in Table 1. The most promising compounds, selected on the basis of their strong antiproliferative effects, were tested for growth inhibition of leukemia L-1210 cells and colon carcinoma SW-948 cells in vitro.

**Table 1:** Activities of 10-substituted 1,8-diazaphenothiazines in selected immunological assays

No.	Cytotoxicity against PBMC		Inhibition of PHA-induced PBMC proliferation			TNF-α inhibition
	10 µg/ml	50 µg/ml	1 µg/ml	10 µg/ml	50 µg/ml	5 µg/ml
**4**	6.7	21.4	5.0	74.4	78.6	50.4
**7**	0.8	1.7	9.6	22.9	45.6	76.4
**8**	−0.3	−6.0	19.0	26.0	55.6	89.3
**9**	−1.1	8.8	9.3	24.4	41.2	87.4
**10**	2.0	2.6	13.6	26.8	45.5	85.9
**12**	6.6	8.1	4.1	5.2	26.2	54.8
**13**	−3.6	15.0	5.7	20.9	81.1	86.7
**14**	−0.7	11.9	1.4	19.2	59.4	89.1
**15**	1.3	12.1	−6.8	−5.4	59.6	75.0
**16**	0.9	10.0	−0.9	−2.9	47.0	85.6
**17**	1.5	7.3	−0.9	−0.5	18.0	47.6
**18**	−1.4	18.7	−3.4	5.1	67.4	73.1
**19**	−4.5	4.8	−0.9	7.0	18.2	46.1
**20**	−2.0	−0.1	3.6	12.5	42.2	76.0
**22**	−5.0	6.7	8.9	16.2	62.5	5.8
**23**	−0.9	12.5	9.4	19.3	50.2	48.6
**24**	−1.6	4.5	8.4	12.4	46.8	7.3

The table shows the degree of cytotoxicity against PBMC, effects on PHA-induced proliferative response of human PBMC and LPS-induced TNF-α production by these cells. The results are given in percentage inhibition as compared with appropriate DMSO controls. Positive values denote inhibition, negative stimulation

The proliferation test was performed at the concentrations of 1, 10, and 50 µg/ml. A strong activity (inhibition over 70 %) was exhibited by compound: **4** at 10 µg/ml and compound **13** at 50 µg/ml in comparison with the control cultures (culture medium containing appropriate dilution of DMSO). These compounds possess the hydrogen atom and dimethylaminopropyl groups at position 10. A moderate activity (inhibition about 60 % at 50 µg/ml) was exhibited by compounds: **14**, **15**, **18,** and **22** (the dimethylamino-2-methylpropyl, diethylaminoethyl, 1-methyl-2-piperidinoethyl, and acetamidopropyl groups). Other compounds were weakly active or inactive.

In order to check whether the inhibitory effects of the compounds were not caused by cytotoxicity, the compounds were tested for their effects on viability of PBMC. All the compounds exhibited very weak cytotoxic properties with the inhibition of cell viability not exceeding 22 % even at 50 μg/ml. Because lack of toxicity at 1 μg/ml that concentration of the compounds was deleted in Table 1.

The compounds were also tested for their inhibitory effects on LPS-induced TNF-α production at the concentrations of 5 and 25 μg/ml. No further inhibition of TNF-α production was registered for 25 μg/ml and, therefore, not shown in Table 1. Compounds **8**–**10**, **13**, **14,** and **16** showed inhibitions of over 85 % at 5 μg/ml.

**Fig. 1:**
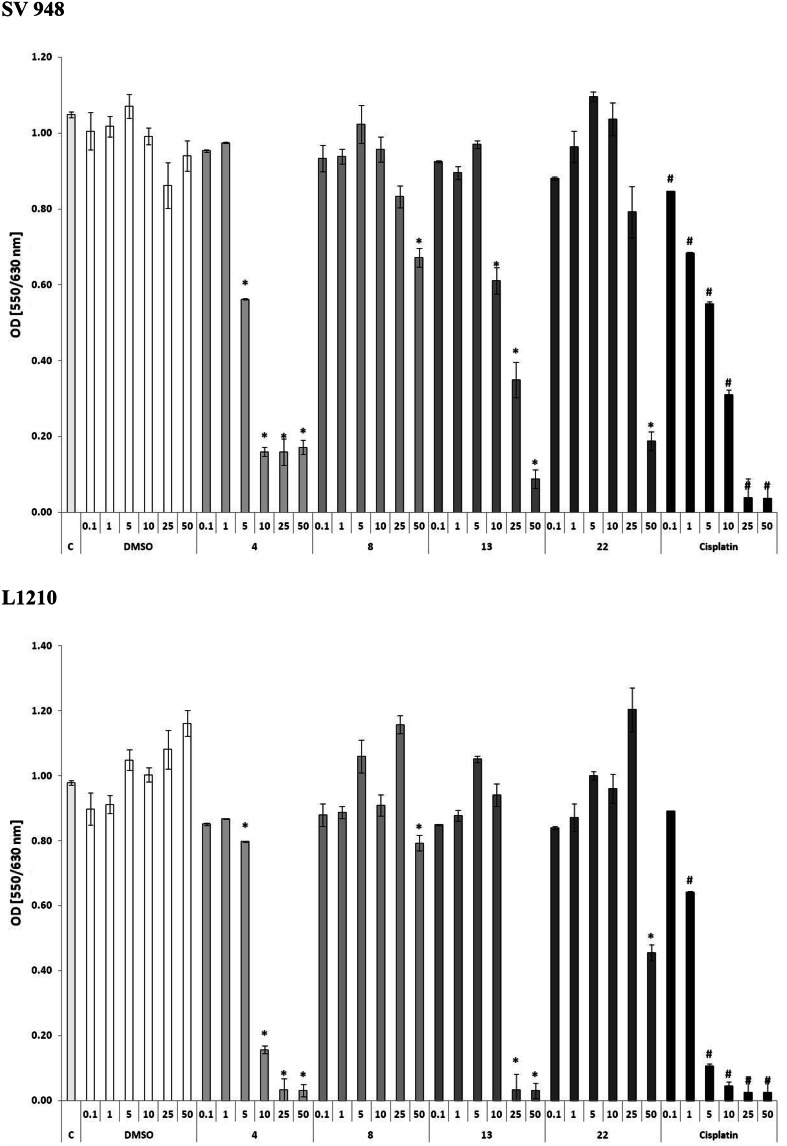
The anticancer activities of selected compounds at concentrations of 0.1–50 µg/ml. L-1210 and SW-948 cell lines were used in the study. The results are presented as the mean optical density ± SE (*versus DMSO; ^#^versus Control, *p* < 0.001)

The most promising compounds **4**, **8**, **13,** and **22** (being strongly antiproliferative and low cytotoxic) were selected for evaluation of anticancer activities against the cancer cell lines at the concentrations of 0.1–50 µg/ml using cisplatin as the reference drug (Fig. 1). The most active was compound **4**, exhibiting similar anticancer activity to cisplatin against colon carcinoma SV-948 cells at the concentration of 5 µg/ml and against leukemia L-1210 cells at 10 µg/ml (Table 2). Compounds **13** and **22** showed strong inhibition at 10 µg/ml. It is worth noting that cisplatin showed high toxicity killing of 50 % of granulocyte/macrophage progenitor cells already at 0.9 μg/ml after 1 h of culture (Umbach *et al.,* 1984). The drug is also nephrotoxic (Yao *et al.,* 2007). The ability of the compounds (in particular **4** and **13**) to strongly inhibit TNF-α may be of additional advantage in anti-tumor therapy. Although TNF-α may have a dual role in tumor progression (Wajant, 2009) some anti-tumor strategies aim at inhibition of its activity (Guadagni *et al.,* 2007).

**Table 2:** Anticancer activity (IC_50_) of selected compounds **4** and **13** and cisplatin as a reference drug against cancer lines SW-948 and L-1210

Compound	IC_50_ (μg/ml)	
	SW-948	L-1210
**4**	5.47	7.41
**13**	14.95	6.03
**Cisplatin**	5.52	2.13

It is interesting that the most active was compound **4**, possessing the hydrogen atom instead of the pharmacophoric aminoalkyl substituents at the thiazine nitrogen atom. It seems that compound **4** displays a different mechanism of action than that found for substituted phenothiazines and azaphenothiazines with the acylaminoalkyl and chloroethylureidoalkyl groups (Motohashi *et al.,* 2000, 2006; Pluta *et al.,* 2011).

## Conclusion

We report here a few step synthesis and biological activity of novel tricyclic 10*H*- and 10-substituted 1,8-diazaphenothiazines. The synthesis was run through the Smiles rearrangement of S–N type. The structure diazaphenothiazine system was elucidated using the NOE experiment and 2D (^1^H–^1^H and ^1^H–^13^C) spectra. Some 1,8-diazaphenothiazines exhibited antiproliferative, anticancer, TNF-α inhibitory activities with low cytotoxicity. The new diazaphenothiazine system was found to be pharmacophoric as 10*H*-1,8-diazaphenothiazine was the most active, with anticancer activities comparable to that of cisplatin. This compound seems to be a useful starting point for further study to found more potent anticancer agents by introduction of new substituents at the thiazine nitrogen atom.

## Experimental

### Chemistry

Melting points were determined in open capillary tubes on a Boetius melting point apparatus and are uncorrected. The ^1^H NMR, COSY, NOE HSQC, HMBC spectra were recorded on a Bruker Fourier 300 and Bruker DRX spectrometers at 300 and 600 MHz in deuteriochloroform with tetramethylsilane as the internal standard. The ^13^C NMR spectrum was recorded at 75 MHz. Electron Impact mass spectra (EI MS) and Fast Atom Bombardment mass spectra (FAB MS, in glycerol) were run on a Finnigan MAT 95 spectrometer at 70 eV. The thin layer chromatography were performed on silica gel 60 F_254_ (Merck 1.05735) with CHCl_3_-EtOH (5:1 and 10:1 v/v) and on aluminum oxide 60 F_254_ neutral (type E) (Merck 1.05581) with CHCl_3_-EtOH (10:1 v/v) as eluents.

#### Synthesis of 10*H*-1,8-diazaphenothiazine (**4**)

##### From sodium 3-amino-4-pyridinethiolate (**1**) and 2-chloro-3-nitropyridine (**2**)

To a solution of 148 mg (1 mmol) sodium 3-amino-4-pyridinethiolate (**1**) in 10 ml dry DMF was added 158 mg (1 mmol) 2-chloro-3-nitropyridine (**2**). The mixture was stirred at rt 3 h and next was refluxed 3 h. After cooling, the reaction mixture was evaporated in vacuo. The dry residue was dissolved in CHCl_3_ and purified by column chromatography (aluminum oxide, CHCl_3_) to give

- 10*H*-1,8-diazaphenothiazine (**4**) (0.125 g, 62 %) mp 135–136 °C.

^1^H NMR (CDCl_3_) *δ* 6.73 (dd, *J* = 7.5 Hz, *J* = 5.1 Hz, 1H, H_3_), 6.84 (d, *J* = 5.0 Hz, 1H, H_6_), 7.11 (dd, *J* = 7.5 Hz, *J* = 1.5 Hz, 1H, H_4_), 7.69 (board s, 1H, N–H), 7.84 (dd, *J* = 5.1 Hz, *J* = 1.5, 1H, H_2_), 7.89 (s, 1H, H_9_), 7.95 (d, *J* = 5,0 Hz, 1H, H_7_). ^13^C NMR (CDCl_3_) *δ* 112.2 (C_4a_), 118.9 (C_3_), 120.5 (C_6_), 128.9 (C_5a_), 134.3 (C_4_), 134.4 (C_9_), 136.9 (C_9a_), 143.1 (C_7_), 145.9 (C_2_), 152.1 (C_10a_). EI MS *m*/*z*: 201 (M, 100), 174 (M-HCN, 30). Anal. Calcd for: C_10_H_7_N_3_S, C 59.68, H 3.51, N 20.88; S 15.93. Found: C 59.49, H 3.53, N 20.80; S 15.79.

(b) 3-amino-3′-nitro-2,4′-dipyridinyl sulfide (**5**) (0.025 g, 9 %) mp 147–148 °C.

##### In cyclization of 3-amino-3′-nitro-2,4′-dipyridinyl sulfide (**5**)

The brown solution of 124 mg (0.5 mmol) 3-amino-3′-nitro-2,4′-dipyridinyl sulfide **5** in 5 ml dry DMF was refluxed for 4 h. After cooling, the reaction mixture was evaporated in vacuo. The dry residue was dissolved in CHCl_3_ and purified by column chromatography (aluminum oxide, CHCl_3_) to give 10*H*-1,8-diazaphenothiazine (**4**) (0.088 g, 88 %)

#### Synthesis of 10-substituted 1,8-diazaphenothiazines **7**, **8**, and **10**–**12**

To a solution of 10*H*-1,8-diazaphenothiazine (**4**) (0.100 g, 0.5 mmol) in dry DMF (5 ml) NaH (0.024 g, 1 mmol, 60 % NaH in mineral oil was washed out with hexane) was added. The reaction mixture was stirred at room temperature for 1 h and then alkyl, aryl, and heteroaryl halides (methyl iodide, allyl bromide, benzyl chloride, 1-fluoro-4-nitrobenzene, 4-chloro-3-pyridine, 1.5 mmol) were added and the stirring was continued for 24 h. The mixture was poured into water (15 ml), extracted with CHCl_3_ (3 × 10 ml), and dried using Na_2_SO_4_. The obtained product was purified by column chromatography (aluminum oxide, CHCl_3_) to give

##### 10-Methyl-1,8-diazaphenothiazine (**7**) (0.085 g, 79 %); mp 82–83 °C

^1^H NMR (CDCl_3_) *δ* 3.44 (s, 3H, CH_3_), 6.90 (dd, *J* = 7.2 Hz, *J* = 4.9 Hz, 1H, H_3_), 7.18 (d, *J* = 5.4 Hz, 1H, H_6_), 7.26 (dd, *J* = 7.8 Hz, *J* = 1.8 Hz, 1H, H_4_), 7.90 (s, 1H, H_9_), 8.07 (d, *J* = 5.4 Hz, 1H, H_7_), 8.09 (dd, *J* = 4.9 Hz, *J* = 1.8 Hz, 1H, H_2_). ^13^C NMR (CDCl_3_) *δ* 32.8 (NCH_3_), 115.0 (C_4a_), 118.2 (C_3_), 120.8 (C_6_), 131.9 (C_5a_), 134.4 (C_4_), 135.2 (C_9_), 139.9 (C_9a_), 143.9 (C_7_), 145.8 (C_2_), 154.3 (C_10a_). EI MS *m*/*z*: 215 (M, 100), 200 (M-CH_3_, 80). Anal. Calcd for: C_11_H_9_N_3_S C 61.37, H 4.21, N 19.52. Found: C 61.22; H 4.23; N 19.41.

##### 10-Allyl-1,8-diazaphenothiazine (**8**) (0.085 g, 70 %); an oil

^1^H NMR (CDCl_3_) *δ* 4.66 (m, 2H, N-CH_2_), 5.32 (m, 2H, =CH_2_), 5.96 (m, 1H, CH), 6.82 (dd, *J* = 7.5 Hz, *J* = 5.1 Hz, 1H, H_3_), 7.04 (d, *J* = 5.0 Hz, 1H, H_6_), 7.18 (dd, *J* = 7.5 Hz, *J* = 1.5 Hz, 1H, H_4_), 7.89 (s, 1H, H_9_), 8.02 (m, 2H, H_2,_ H_7)_. ^13^C NMR (CDCl_3_) *δ* 47.6 (NCH_2_), 113.0 (C_4a_), 118.1 (C_3_), 119.2 (C_6_), 121.1 (CH_2_=), 130.2 (C_5a_), 131.2 (C_4_), 134.5 (C_9_), 137.9(–CH=), 138.8 (C_9a_), 140.2 (C_7_), 146.4 (C_2_), 151.9 (C_10a_). EI MS *m*/*z*: 241 (M, 50), 200 (M-CH_2_CHCH_2_, 100). Anal. Calcd for: C_13_H_11_N_3_S C 64.70, H 4.59, N 17.41. Found: 64.58; H 4.58; N 17.31.

##### 10-Benzyl-1,8-diazaphenothiazine (**10**) (0.095 g, 65 %); an oil

^1^H NMR (CDCl_3_) *δ* 5.34 (s, 2H, CH_2_), 6.76 (dd, *J* = 7.2 Hz, *J* = 4.8 Hz, 1H, H_3_), 6.87 (d, *J* = 5.0 Hz, 1H, H_6_), 7.22 (dd, *J* = 7.2 Hz, *J* = 1.4 Hz, 1H, H_4_), 7.29 (m, 5H, C_6_H_5_), 7.81 (s, 1H, H_9_), 7.96 (m, 2H, H_2_, H_7_). EI MS *m*/*z*: 291 (M, 80), 200 (M-CH_2_C_6_H_5_, 100). Anal. Calcd for: C_17_H_13_N_3_S C 70.08, H 4.50, N 14.42. Found: C 70.00; H 4.52; N 14.29.

##### 10-(4′-Nitrophenyl)-1,8-diazaphenothiazine (**11**) (0.120 g, 74 %); mp 171–172 °C

^1^H NMR (CDCl_3_) *δ* 6.88 (dd, *J* = 7.2 Hz, *J* = 5.0 Hz, 1H, H_3_), 6.95 (d, *J* = 5.0 Hz, 1H, H_6_), 7.21 (dd, *J* = 7.2 Hz, *J* = 1.6 Hz, 1H, H_4_), 7.55 (m, 2H, 2H C_6_H_4_), 7.81 (dd, *J* = 5.0 Hz, *J* = 1.6 Hz, 1H, H_2_), 7.96 (d, *J* = 5.0 Hz, 1H, H_7_), 8.15 (s, 1H, H_9_), 8.50 (m, 2H, 2H C_6_H_4_). EI MS *m*/*z*: 322 (M, 100), 276 (M-NO_2_, 30), 200 (M-NO_2_C_6_H_4_, 18). Anal. Calcd for: C_16_H_10_N_4_O_2_S C 59.62, H 3.13, N 17.38. Found: C 59.44; H 3.12; N 17.29.

##### 10-(3′-Nitro-4′-pyridinyl)-1,8-diazaphenothiazine (**12**) (0.130 g, 80 %); mp 189–190 °C

lit. (Morak-Młodawska *et al.,* 2012) mp 189–190 °C.

#### Synthesis of 10-propargyl-1,8-diazaphenothiazines (**9**)

To a suspension of 100 mg (0.5 mmol) 10*H*-1,8-diazaphenothiazine (**4**) in 10 ml DMF was added 80 mg (0.72 mmol) potassium *tert*-butoxide. The mixture was stirred at room temperature for 1 h. Then to the solution was added dropwise a solution of propargyl bromide 80 mg (0.64 mmol) in toluene. The solution stirred at room temperature for 24 h and poured onto water (20 ml), extracted with methylene chloride (20 ml), dried with Na_2_SO_4_, and evaporated to the brown oil. The residue was purified by column chromatography (silica gel, CHCl_3_) to yield 85 mg (71 %) of 10-propargyl-1,8-diazaphenothiazine (**9**), mp 96–97 °C.

^1^H NMR: *δ* 2.39 (t, *J* = 2.5 Hz, 1H), 4.61 (t, *J* = 2.5 Hz, 2H), 6.92 (dd, *J* = 7.5 Hz, *J* = 5.1 Hz 1H, H_3_), 7.23 (m, 2H, H_4_, H_6_), 8.10 (d, *J* = 5.5 Hz, 1H, H_7_), 8.13 (s,1H, H_9_), 8.15 (dd, *J* = 5.1 Hz, *J* = 1.3 Hz, 1H, H_2_). EI MS: 239 (M, 100), 200 (M-CH_2_CCH, 85). Anal. Calcd for: C_13_H_9_N_3_S C 65.25, H 3.79, N 17.56. Found: C 65.20; H 3.77; N 17.39.

#### Synthesis of 10-substituted 1,8-diazaphenothiazines **13**–**19**

To a solution of 10*H*-1,8-diazaphenothiazine (**4**) (0.100 g, 0.5 mmol) in dry dioxane (10 ml) NaOH (0.20 g, 5 mmol) was added. The mixture was refluxed 1 and 5 h then the hydrochlorides of dialkylaminoalkyl chloride (3-dimethylaminopropyl, 2-diethylaminoethyl, 3-dimethylamino-2-methylpropyl) and hydrochlorides of cycloaminoethyl chloride (*N*-(2-chloroethyl)-pyrrolidine, 2-(1-methyl-2′-piperydinyl)ethylchloride, *N*-(2-chloroethyl)piperidine, *N*-(2-chloroethyl)morpholine, 1.5 mmol) were added. The reaction mixture was refluxed for 24 h. After cooling, dioxane was evaporated in vacuo and residue was dissolved in CHCl_3_ (10 ml). The extracts were washed with water, dried with anhydrous sodium sulfate, and evaporated in vacuo. The obtained product was purified by column chromatography (aluminum oxide, CHCl_3_-EtOH 10:1) to give

##### 10-(3′-Dimethylaminopropyl)-1,8-diazaphenothiazine (**13**) (0.100 g, 70 %); an oil

^1^H NMR: *δ* 2.00 (m, 2H, CH_2_), 2.26 (s, 6H, 2CH_3_), 2.44 (t, *J* = 7.5 Hz, 2H, NCH_2_), 4.10 (t, *J* = 7.5 Hz, 2H, NCH_2_), 6.73 (m, 1H, H_3_), 6.89 (d, *J* = 4.8 Hz, 1H, H_6_), 7.16 (d, *J* = 7.2 Hz, 1H, H_4_), 7.99 (m, 2H, H_2_, H_7_), 8.08 (s, 1H, H_9_). ^13^C NMR (CDCl_3_) *δ* 24.2 (CH_2_), 42.9 (CH_2_), 45.5 (N(CH_3_)_2_), 57.13 (CH_2_), 114.6 (C_4a_), 118.1 (C_3_), 120.8 (C_6_), 131.8 (C_5a_), 134.7 (C_4_), 135.5 (C_9_), 138.7 (C_9a_), 143.6 (C_7_), 145.6 (C_2_), 153.6 (C_10a_). FAB MS *m*/*z*: 287 (M+1, 100), 202 (M+1-C_3_H_6_NC_2_H_6_, 19). Anal. Calcd for C_15_H_18_N_4_S C 62.91; H 6.33; N 19.56. Found: C 62.78; H 6.30; N 19.39.

##### 10-(3′-Dimethylamino-2′-methylpropyl)-1,8-diazaphenothiazine (**14**) (0.125 g, 83 %); an oil

^1^H NMR: *δ* 1.02 (d, *J* = 6.5 Hz, 3H, CH_3_), 2.39 (m, 9H, 2CH_3,_ CH_2_, CH), 4.15 (m, 2H, CH_2_), 6.80 (dd, *J* = 7.4 Hz, *J* = 5.2 Hz, 1H, H_3_), 6.85 (d, *J* = 5.0 Hz, 1H, H_6_), 7.20 (dd, *J* = 7.4 Hz, *J* = 1.4 Hz, 1H, H_4_), 8.02 (dd, *J* = 5.2 Hz, *J* = 1.4 Hz, 1H, H_2_), 8.09 (d, *J* = 5.0 Hz, 1H, H_7_), 8.15 (s, 1H, H_9_). FAB MS *m*/*z*: 301 (M+1, 100), 202 (M+1-C_2_H_4_NC_4_H_10_, 18). Anal. Calcd for: C_16_H_20_N_4_S C 63.97; H 6.71; N 18.65. Found: C 63.80; H 6.73; N 18.42.

##### 10-(2′-Diethylaminoethyl)-1,8-diazaphenothiazine (**15**) (0.113 g, 75 %); an oil

^1^H NMR: *δ* 1.04 (t, *J* = 7.3 Hz, 6H, 2CH_3_), 2.62 (q, *J* = 7.3 Hz, 4H, 2CH_2_), 3.62 (t, *J* = 7.4 Hz, 2H, CH_2_), 4.15 (t, *J* = 7.4 Hz, 2H, CH_2_), 6.76 (dd, *J* = 7.2 Hz, *J* = 5.1 Hz, 1H, H_3_), 6.83 (d, *J* = 5.0 Hz, 1H, H_6_), 7.16 (dd, *J* = 7.2 Hz, *J* = 1.2 Hz, 1H, H_4_), 7.96 (dd, *J* = 5.1 Hz, *J* = 1.6 Hz, 1H, H_2_), 8.03 (d, *J* = 5.0 Hz, 1H, H_7_), 8.09 (s, 1H, H_9_). FAB MS *m*/*z*: 301 (M+1, 100), 202 (M+1-C_2_H_4_NC_4_H_10_, 25). Anal. Calcd for: C_16_H_20_N_4_S C 63.97; H 6.71; N 18.65. Found: C 63.81; H 6.73; N 18.41.

##### 10-(2′-Pyrrolidinylethyl)-1,8-diazaphenothiazine (**16**) (0.110 g, 75 %); an oil

^1^H NMR (CDCl_3_) *δ* 1.90 (m, 4H, 2CH_2_), 2.72 (m, 4H, 2CH_2_), 3.09 (t, *J* = 7.2 Hz, 2H, CH_2_), 4.35 (t, *J* = 7.2 Hz, 2H, NCH_2_), 6.70 (dd, *J* = 7.6 Hz, *J* = 5.0 Hz, 1H, H_3_), 6.83 (d, *J* = 5.0 Hz, 1H, H_6_), 7.17 (dd, *J* = 7.2 Hz, *J* = 1.5 Hz 1H, H_4_), 7.97 (dd, *J* = 5.0 Hz, *J* = 1.5 Hz, 1H, H_2_), 8.04 (d, *J* = 5.0 Hz, 1H, H_7_), 8.19 (s, 1H, H_9_). FAB MS *m*/*z*: 299 (M+1, 100), 202 (M+1-C_2_H_4_NC_4_H_8_, 29). Anal. Calcd for: C_16_H_18_N_4_S C 64.40; H 6.08; N 18.78. Found: C 64.25; H 6.05; N 18.55.

##### 10-(2′-Piperydinylethyl)-1,8-diazaphenothiazine (**17**) (0.110 g, 70 %); an oil

^1^H NMR (CDCl_3_) *δ* 1.47 (m, 2H, CH_2_),1.63 (m, 4H, 2CH_2_) 2.54 (m, 4H, 2CH_2_), 2.75 (t, *J* = 6.8 Hz, 2H, CH_2_), 4.22 (t, *J* = 6.8 Hz, 2H, NCH_2_), 6.73 (dd, *J* = 7.6 Hz, *J* = 5.0 Hz, 1H, H_3_), 6.85 (d, *J* = 5.0 Hz, 1H, H_6_), 7.14 (dd, *J* = 7.6 Hz, *J* = 1.6 Hz 1H, H_4_), 7.97 (dd, *J* = 5.0 Hz, *J* = 1.6 Hz, 1H, H_2_), 8.03 (d, *J* = 5.0 Hz, 1H, H_7_), 8.18 (s, 1H, H_9_). FAB MS *m*/*z*: 313 (M+1, 100), 202 (M+1-C_2_H_4_NC_5_H_10_, 20). Anal. Calcd for: C_17_H_20_N_4_S: C 65.35; H 6.45; N 17.93. Found: C 65.22; H 6.47; N 17.80.

##### 10-(1′-Methyl-2′-piperydinylethyl)-1,8-diazaphenothiazine (**18**) (0.116 g, 72 %); an oil

^1^H NMR (CDCl_3_) *δ* 1.30–2.15 (m, 7H_)_, 2.36 (s, 3H, NCH_3_), 2.85 (m, 1H, CH), 4.0 (m, 2H, NCH_2_), 6.73 (dd, *J* = 7.6 Hz, *J* = 5.1 Hz, 1H, H_3_), 6.87 (d, *J* = 5.0 Hz, 1H, H_6_), 7.14 (dd, *J* = 7.6 Hz, *J* = 1.6 Hz, 1H, H_4_), 7.97 (dd, *J* = 5.1 Hz, *J* = 1.6 Hz, 1H, H_2_), 8.03 (d, *J* = 5.0 Hz, 1H, H_7_), 8.06 (s, 1H, H_9_). FAB MS. 327 (M+H, 80), 313 (M+1-CH_3_ 100). Anal. Calcd for: C_18_H_22_N_4_S C 66.22; H 6.79; N 17.16. Found: C 66.17; H 6.75; N 17.03.

##### 10-(2′-Morpholinylethyl)-1,8-diazaphenothiazine (**19**) (0.106 g, 68 %); an oil

^1^H NMR (CDCl_3_) *δ* 1.67 (m, 4H, 2CH_2_), 2.59 (m, 4H, 2CH_2_), 2.82 (t, *J* = 6.6 Hz, 2H, CH_2_), 4.22 (t, *J* = 6.6 Hz, 2H, NCH_2_) 6.75 (dd, *J* = 7.2 Hz, *J* = 5.1 Hz, 1H, H_3_), 6.88 (d, *J* = 5.0 Hz, 1H, H_6_), 7.15 (dd, *J* = 7.6 Hz, *J* = 1.6 Hz 1H, H_4_), 7.97 (dd, *J* = 5.1 Hz, *J* = 1.6 Hz, 1H, H_2_), 8.03 (d, *J* = 5.0 Hz, 1H, H_7_), 8.16 (s, 1H, H_9_). FAB MS *m*/*z*: 315 (M+1, 40), 202 (M+1-C_2_H_4_NOC_4_H_8_, 15), 114 (C_2_H_4_NC_5_H_10_, 100). Anal. Calcd for: C_16_H_18_N_4_OS: C 61.12; H 5.77; N 17.82. Found: C 61.03; H 5.73; N 17.68.

#### Synthesis of 10-phthalimidopropyl-1,8-diazaphenothiazines (**20**)

To a stirred solution of 10*H*-1,8-diazaphenothiazine (**4**) (0.100 g, 0.5 mmol) in dry toluene (20 ml) NaH (0.12 g, 5 mmol, washed out with hexane) was added. The mixture was stirred for 20 min at rt, then refluxed for 1 h and a solution of 1.5 mmol, *N*-(3-bromopropyl) phthalimide 0.405 g, in toluene (10 ml) was added. The mixture was refluxed for 24 h. After cooling, the resulted solid was filtered off, toluene was evaporated in vacuo and the residue was purified by column chromatography (aluminum oxide, CHCl_3_) to give 10-(3′-phthalimidopropyl)-1,8-diazaphenothiazine (**20**) (0.110 g, 70 %), mp 40–41 °C.

^1^H NMR (CDCl_3_) *δ* 2.39 (m, 2H, CH_2_), 3.86 (t, *J* = 6.1 Hz, 2H, NCH_2_), 4.13 (t*, J* = 6.0 Hz, 2H, NCH_2_), 6.77 (dd, *J* = 7.1 Hz, *J* = 4.9 Hz Hz, 1H, H_3_), 6.88 (d, *J* = 5.0 Hz, 1H, H_6_), 7.14 (dd, *J* = 7.1 Hz, *J* = 1.4 Hz, 1H, H_4_), 7.71 (m, 2H_phthalimide_), 7.79 (dd, dd, *J* = 4.9 Hz, *J* = 1.4 Hz, 1H, H_2_), 7.82 (m, 2H_phthalimide_), 7.98 (s, 1H, H_9_), 8.07 (d, *J* = 5.0 Hz, 1H, H_7_). FAB MS *m*/*z*: 389 (M+H, 100), 201 (M+1-(CH_2_)_3_N(CO)_2_C_6_H_4_, 30). Anal. calcd. For C_21_H_16_N_4_O_2_S: C 64.93, H 4.15, N 14.42. Found: C 64.82; H 4.14; N 14.29.

#### Hydrolysis of 10-phthalimidopropyl-1,8-diazaphenothiazine (**20**)

To a solution of 10-phthalimidopropyl-1,8-diazaphenothiazine (**20**) (0.388 g, 1 mmol) in EtOH (20 ml) 80 % aqueous solution of hydrazine (0.2 ml, 5 mmol) was added. The mixture was refluxed for 2 h. After cooling, the reaction mixture was acidified with conc. hydrochloric acid to pH 2. The solution was concentrated and the resulted solid was filtered off. The filtrate was alkalized with 10 % aqueous solution of sodium hydroxide and extracted with CHCl_3_ (3 × 10 ml). The extracts were washed with water, dried with anhydrous sodium sulfate, and evaporated in vacuo. The obtained residue with 10-aminopropyl-1,8-diazaphenothiazine (**21**) was fast used in the synthesis of the amide derivatives of 1,8-diazaphenothiazines (**22**–**24**).

#### Synthesis of 10-(3′-acetamidopropyl)-1,8-diazaphenothiazine (**22**)

To a suspension with the oil of 10-aminopropyl-1,8-diazaphenothiazine (**21**)(0.129 g, 0.5 mmol) in pyridine (5 ml) acetic anhydride (1.48 ml, 1.5 mmol) was added and the mixture was stirred at rt for 2 h. After evaporation of pyridine in vacuo the residue was dissolved in CHCl_3_ (10 ml). The solution was washed with water, dried with anhydrous sodium sulfate, and evaporated in vacuo. The residue was purified by column chromatography (aluminum oxide, CHCl_3_) to give 0.120 g (80 %) 10-(3′-acetamidopropyl)-2,7-diazaphenothiazine (**22**), mp 120 °C.

^1^H NMR (CDCl_3_) *δ* 2.05 (s, 3H, CH_3_), 2.07 (m, 2H, CH_2_), 3.44 (m, 2H, NCH_2_), 3.96 (t, *J* = 6.6 Hz, 2H, NCH_2_), 5.99 (broad s, 1H, NH), 6.73 (dd, *J* = 7.2 Hz, *J* = 5.0 Hz, 1H, H_3_), 6.85 (d, *J* = 5.0 Hz, 1H, H_6_), 7.14 (dd, *J* = 7.2 Hz, *J* = 1.4 Hz, 1H, H_4_), 7.97 (dd, *J* = 5.0 Hz, *J* = 1.4 Hz 1H, H_2_), 8.03 (d, *J* = 5.0 Hz, 1H, H_7_), 8.18 (s, 1H, H_9_). FAB MS *m*/*z*: 301 (M+H, 100), 202 (M+1–C_3_H_5_NHCOCH_3_, 30). Anal. calcd. For C_15_H_16_N_4_OS: C 59.98; H 5.37; N 18.65. Found: C 59.83; H 5.35; N 18.55.

#### Synthesis of 10-(3′-methanesulfonamidopropyl)-1,8-diazaphenothiazine (**23**)

To a stirred solution of oil with 10-aminopropyl-1,8-diazaphenothiazine (**21**) (0.129 g, 0.5 mmol) in a mixture of CH_2_Cl_2_ (5 ml) and 10 % aqueous Na_2_CO_3_ solution (5 ml) a solution of methanesulfonyl chloride (0.12 ml, 1.5 mmol) in CH_2_Cl_2_ (3 ml) was added. The solutions were stirred at rt for 24 h. The organic phase was separated and aqueous phase was extracted with CH_2_Cl_2_ (2 × 5 ml). The combined extracts were washed with water (10 ml) and dried with anhydrous sodium sulfate and evaporated in vacuo. The residue was purified by column chromatography (aluminum oxide, CH_2_Cl_2_) to give 0.125 g (74 %) 10-(3′-methanesulfonamidopropyl-1,8-diazaphenothiazine (**23**) as an oil.

^1^H NMR (CDCl_3_) *δ* 2.08 (m, 2H, CH_2_), 2.94 (s, 3H, CH_3_), 3.42 (m, 2H, NCH_2_), 4.02 (t, *J* = 6.9 Hz, 2H, NCH_2_), 5.57 (broad s, 1H, NH), 6.74 (dd, *J* = 7.2 Hz, *J* = 5.0 Hz, 1H, H_3_), 6.84 (d, *J* = 5.0 Hz, 1H, H_6_), 7.14 (dd, *J* = 7.2 Hz, *J* = 1.4 Hz 1H, H_4_), 7.97 (dd, *J* = 5.0 Hz, *J* = 1.4 Hz 1H, H_2_), 8.03 (d, *J* = 5.0 Hz, 1H, H_7_), 8.18 (s, 1H, H_9_). FAB MS *m*/*z*: 337 (M+1, 100), 202 (M+1-C_3_H_5_NHSO_2_CH_3_,30). Anal. calcd. For C_14_H_16_N_4_O_2_S_2_: C 49.98; H 4.79; N 16.65. Found: C 49.88; H 4.74; N 16.39.

#### Synthesis of 10-(3′-chloroethylureidopropyl)-1,8-diazaphenothiazine (**24**)

To a stirred solution of 10-aminopropyl-1,8-diazaphenothiazine (**21**) (0.129 g, 0.5 mmol) in dry EtOH (10 ml) at 0 °C 2-chloroethyl isocyanate (0.87 ml, 1 mmol) was added. The mixture was stirred at 0 °C for 0.5 h and at rt for 24 h. After evaporation of EtOH in vacuo the residue was purified by column chromatography (aluminum oxide, CH_2_Cl_2_) to give 0.120 g (63 %) 10-chloroethylureidopropyl-1,8-diazaphenothiazine (**24**), mp 103 °C.

^1^H NMR (CDCl_3_) *δ* 1.75 (m, 2H, CH_2_), 2.10 (m, 2H, CH_2_), 3.49 (m, 4H, 2CH_2_), 4.46 (m, 2H, CH_2_), 6.76 (dd, *J* = 7.2 Hz, *J* = 5.1 Hz, 1H, H_3_), 6.84 (d, *J* = 5.0 Hz, 1H, H_6_), 7.14 (dd, *J* = 7.2 Hz, *J* = 1.4 Hz 1H, H_4_), 7.96 (dd, *J* = 5.1 Hz, *J* = 1.4 Hz 1H, H_2_), 8.01 (d, *J* = 5.0 Hz, 1H, H_7_), 8.17 (s, 1H, H_9_). FAB MS *m*/*z*: 364 (M+1, 30), 202 (M+H-C_3_H_6_NHCONHCH_2_CH_2_Cl, 10), 185 (2gly + H, 100). Anal. calcd. for C_16_H_18_ClN_5_OS: C 52.82, H 4.99, N 19.25. Found: C 52.77; H 4.97; N 19.11.

### Biological assays

#### Preparation of the compounds for biological assays

The compounds were dissolved in DMSO (10 mg/ml) and subsequently diluted in RPMI-1640 cell culture medium (see below).

#### Isolation of the peripheral blood mononuclear cells

Venous blood from a single donor was withdrawn into heparinized syringes and diluted twice with phosphate-buffered saline. PBMC were isolated by centrifugation on Ficoll-uropoline gradient (density 1.077 g/ml) and centrifuged at 800×*g* for 20 min at 4 °C. The interphase cells, consisting of lymphocytes (20 %) and monocytes (80 %) were then washed three times with Hanks’ medium and re-suspended in a culture medium, referred to below as the culture medium, consisting of RPMI-1640, supplemented with 10 % fetal calf serum, l-glutamine, sodium pyruvate, 2-mercaptoethanol, and antibiotics, at density of 2 × 10^6^ cells/ml.

#### PHA-induced proliferation of human blood mononuclear cells

The isolated PBMC were distributed into 96-well flat-bottom plates in 100 µL aliquots (2 × 10^5^ cells/well). PHA was added at a concentration of 5 µg/ml. The compounds were tested at doses of 1, 10, and 50 µg/ml. DMSO at appropriate dilutions served as control. After a four-day incubation in a cell culture incubator, the proliferative response of the cells was determined by the colorimetric MTT method (Hansen *et al.,* 1989). The results are given in percentage inhibition as compared with appropriate DMSO controls.

#### Cytotoxicity of the compounds against human blood mononuclear cells

PBMC at density of 2 × 10^5^/well, re-suspended in the culture medium, were cultured for 24 h in a cell culture incubator with the preparations at indicated concentrations. Cell survival was determined by MTT colorimetric method (Hansen *et al.,* 1989). The results are given in percentage inhibition as compared with appropriate DMSO controls.

#### Lipopolysaccharide-induced TNF-a production in whole blood cell culture

Venous blood from a single donor was diluted 10× with RPMI-1640 medium and distributed in 1 ml aliquots in 24-well culture plates. The cultures were stimulated by addition of 1 µg/ml of LPS from *E. coli,* O111:B4. The compounds were added to the cultures at concentrations of 5 and 25 µg/ml. Higher concentrations of the compounds could not be used because of inhibitory effects on TNF-α production by corresponding DMSO (the solvent) dilutions. Appropriate dilutions of DMSO served as controls. After overnight incubation in a cell culture incubator, the supernatants were harvested and frozen at −20 °C until cytokine determination by a biological assay (Espevik and Nissen-Meyer, 1986). The results are given in percentage inhibition as compared with appropriate DMSO controls.

#### Growth inhibition of tumor cell lines

L-1210 lymphoma and SW-948 colon tumor cell lines derived from the Collection of Cell Lines of The Institute of Immunology and Experimental Therapy, Wrocław, Poland. The lines were re-suspended in the culture medium and distributed into 96-well flat-bottom plates. L-1210 was present at 1.5 × 10^4^ cells/well while SW-948 and at 2.5 × 10^4^ cells/well. The preparations were added to the wells at the concentration range of 0.1–50 µg/ml. Cisplatin was used as a reference drug in the same concentrations. After 3-day incubation in a cell culture incubator, the proliferation was determined using MTT colorimetric method. The data are presented as a mean OD value from quadruplicate wells ± SE.

#### Statistics

The results are presented as mean values ± standard error (SE) or percentage inhibition = [(control value − tested value)/control value] × 100. Brown-Forsyth’s test was used to determine the homogeneity of variance between groups. When the variance was homogenous, analysis of variance (One-way ANOVA) was applied, followed by post-hoc comparisons with the Tukey’s test to estimate the significance of the difference between groups. Nonparametric data were evaluated with the Kruskal–Wallis’ analysis of variance. Significance was determined at *p* < 0.05. Statistical analysis was performed using STATISTICA 6.1 for Windows.
